# Quantifying Bone Marrow Fat Fraction and Iron by MRI for Distinguishing Aplastic Anemia from Myelodysplastic Syndromes

**DOI:** 10.1002/jmri.27769

**Published:** 2021-06-11

**Authors:** Zhaolong Zeng, Xiangzheng Ma, Yifan Guo, Baodong Ye, Maosheng Xu, Wei Wang

**Affiliations:** ^1^ Radiology Department The First Affiliated Hospital of Zhejiang Chinese Medical University Hangzhou China; ^2^ Radiology Department The First Clinical Medical College of Zhejiang Chinese Medical University, Hangzhou, China Hangzhou China; ^3^ Hematology Department The First Affiliated Hospital of Zhejiang Chinese Medical University Hangzhou China

**Keywords:** aplastic anemia, myelodysplastic syndrome, magnetic resonance quantitation, bone marrow fat, iron

## Abstract

**Background:**

Bone marrow of patients with aplastic anemia (AA) is different from that of patients with myelodysplastic syndrome (MDS) and is difficult to identify by blood examination. IDEAL‐IQ (iterative decomposition of water and fat with echo asymmetry and least‐squares estimation) imaging might be able to quantify fat fraction (FF) and iron content in bone tissues.

**Purpose:**

To determine if IDEAL‐IQ measurements of bone marrow FF and iron content can distinguish between patients with AA and MDS.

**Study Type:**

Retrospective.

**Population:**

Fifty‐seven patients with AA, 21 patients with MDS, and 24 healthy controls.

**Field Strength/Sequence:**

3.0 T, IDEAL‐IQ sequence.

**Assessment:**

Three independent observers evaluated the IDEAL‐IQ images and measured FF and R2* in the left posterior superior iliac spine.

**Statistical Tests:**

Kruskal–Wallis test, linear correlations, and Bland–Altman analysis were used. A *P*‐value of <0.05 was considered statistically significant.

**Results:**

The FF in patients with AA (79.46% ± 15.00%) was significantly higher than that in patients with MDS (42.78% ± 30.09%) and control subjects (65.50% ± 14.73%). However, there was no significant difference in FF between control subjects and patients with MDS (*P* = 0.439). The R2* value of AA, MDS, and controls was 145.38 ± 53.33, (171.13 ± 100.89, and 135.99 ± 32.41/second, respectively, with no significant difference between the three groups (*P* = 0.553).

**Data Conclusion:**

Quantitative IDEAL‐IQ magnetic resonance imaging may facilitate the diagnosis of AA and distinguish it from MDS.

**Level of Evidence:**

3

**Technical Efficacy Stage:**

2

## Introduction

Aplastic anemia (AA) is characterized by microenvironmental changes and bone marrow hypocellularity, resulting in severe pancytopenia and bone marrow failure.^1^ In terms of pathophysiology, the hematopoietic cells in AA are replaced by adipose tissue.^2^ Mesenchymal stem cells from patients with AA are more readily induced to differentiate from adipocytes as opposed to marrow tissue.^3^, ^4^ Bone marrow biopsy and histology of AA have shown hypocellularity, with increased fat content.^5^ The basic diagnostic feature of AA is extensive fatty bone marrow.^6^

On the other hand, myelodysplastic syndromes (MDS) are myeloid neoplasms characterized by clonal proliferation of hematopoietic stem cells, recurrent genetic abnormalities, myelodysplasia, ineffective hematopoiesis, and low count of the peripheral blood cell.^7^ Clinical diagnosis is based on an examination of blood and bone marrow showing low count of the blood cell in at least one hematologic cell line and hypercellular marrow with dysplasia, without an excess of fat cells.^8^ The differential diagnosis of the nonsevere subtype of AA and hypocellular MDS is difficult according to clinical findings or examination of blood.^9^ Routine diagnosis is mostly invasive including bone marrow aspiration to detect morphologic dysplasia and blasts, bone marrow biopsy to assess marrow cellularity and fibrosis, and conventional cytogenetics to detect nonrandom chromosomal abnormalities.^10^

Iterative decomposition of water and fat with echo asymmetry and least‐squares estimation quantitation (IDEAL‐IQ) of complex‐based fat–water R2* magnetic resonance imaging (MRI) has been used to estimate fat fraction (FF).^11^ In IDEAL‐IQ, images are acquired at multiple echo times (TE), and an iterative least‐squares decomposition algorithm is employed to simultaneous solve for FF, water fraction, and R2*.^11^, ^12^ IDEAL‐IQ imaging enables adjustment for common biases in the measurement of tissue fat, including T1 and T2* effects, as well as main magnetic field inhomogeneity.^13^, ^14^ Recently, it has been used in many diagnostic studies. It has been an effective method for noninvasive assessment of pancreatic fat infiltration in diabetic pigs, showing that pancreatic fat infiltration is significantly correlated with diabetes.^15^ IDEAL‐IQ imaging has also been used for quantitative evaluation of changes in vertebral microvascular permeability and vertebral fat deposition in sacroiliac alloxan‐induced diabetic rabbits, which are significantly higher than that of the normal rabbits.^16^ Furthermore, it has also shown that the FF of inactive sacroiliitis was joint or of active sacroiliac arthritis. IDEAL‐IQ imaging has also been used to quantitatively evaluate sacroiliitis in patients with ankylosing spondylitis.^17^

In addition to investigating FF, some studies have used the iron (R2*) images of IDEAL‐IQ to accurately measure the hepatic iron concentration.^18^, ^19^, ^20^ R2* can also reflect iron deposition in bone marrow.^21^, ^22^ IDEAL‐IQ imaging allows for quantification of the FF and simultaneous R2* estimation in one acquisition. Furthermore, it also provides anatomical evaluation of the bone within a very short imaging time^23^, ^24^ and it has been shown that FF and R2* of vertebrae and femur are significantly different.^25^

Thus, the aim of this study was to determine if IDEAL‐IQ MRI measurements of bone marrow FF and iron content can distinguish between patients with AA and MDS.

## Materials and Methods

### Study Population

Our institutional review board approved this retrospective study and the need for written informed consent was waived. From January 2016 to November 2020, patients diagnosed with AA or MDS by pathological bone marrow biopsy of the right posterior superior iliac spine in our hospital were examined using the IDEAL‐IQ sequence. All patients included were older than 18 years. Patients were excluded if they had undergone bone marrow transplant or had other malignant tumors. A control group of 24 healthy individuals were examined with the same MRI protocol. They had no history of hemopathy or related symptoms and MRI showed normal bone marrow.

### MR Imaging

All MRI examinations were performed on a 3‐T MRI system (Discovery MR750, GE Healthcare) using a 32‐channel phased‐array surface coil. After a conventional three‐plane localizer, transverse T2‐weighted (repeat time [TR] = 2400 msec, TE = 56 msec, field of view [FOV] = 400 mm × 400 mm, slice thickness = 5 mm, matrix = 320 × 256, number of excitations [NEX] = 2), transverse T1‐weighted (TR = 420 msec, TE = 10.3 msec, FOV = 400 mm × 400 mm, slice thickness = 5 mm, matrix = 320 × 256, NEX = 2), and coronal T2‐weighted (TR = 3000 msec, TE = 68 msec, FOV = 400 mm × 400 mm, slice thickness = 4 mm, matrix = 320 × 224, NEX = 2) images were acquired using fast spin‐echo sequences for clinical interpretation. IDEAL‐IQ imaging was then performed with the following scan parameters: transverse orientation, TR = 8.3 msec, TE = 3.9 msec, FOV = 360 mm × 360 mm, slice thickness = 5 mm, turning angle = 3°, echo train length = 3, matrix = 224 × 224, NEX = 1. The scanning time was 1 minute and 31 seconds.

### Image Analysis

**FIGURE 1:**
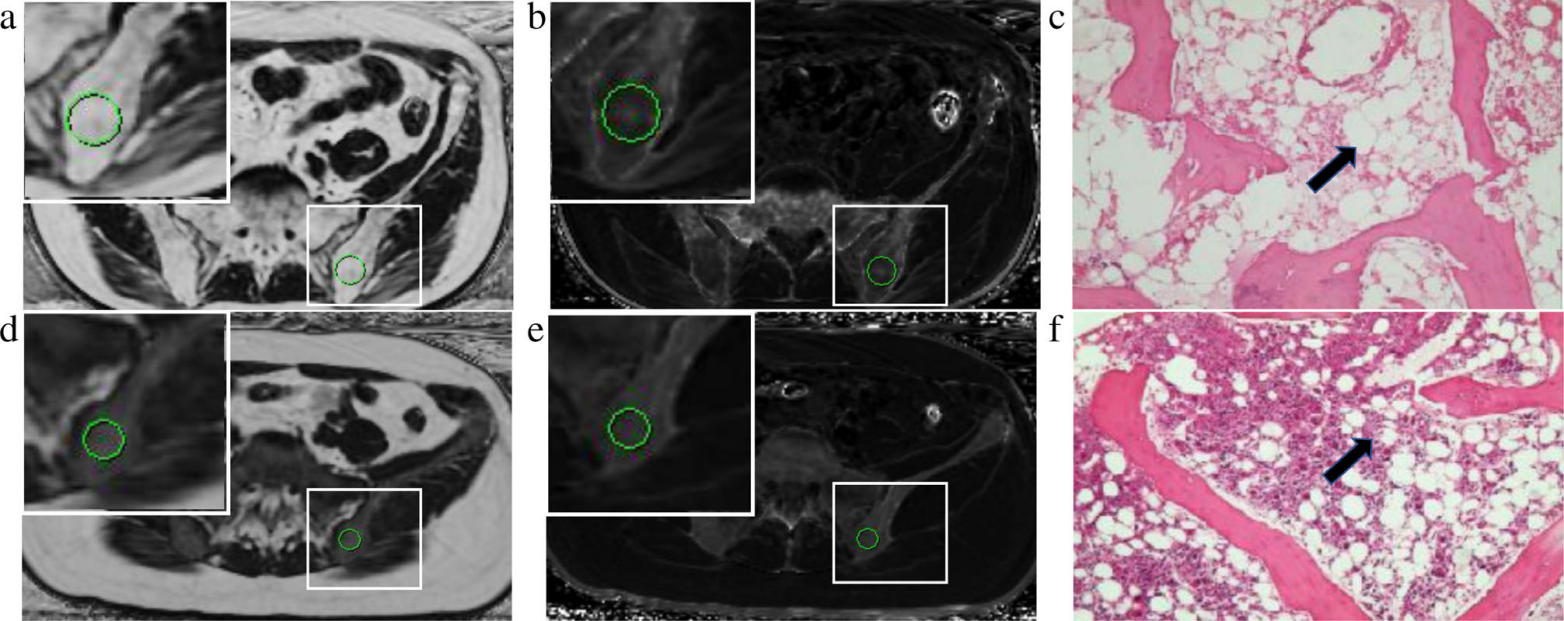
ROIs were manually drawn at the level containing the largest section of the left posterior superior ilium (A, B, D, E). The zoomed version of the ROIs showed in the upper left corner. (A, D) FF value of AA and MDS is 86.98% and 25.84%, respectively. (B, E) Iron content from R2*image is 117.91 and 147.98/second. (C, F) Histologic sections images of AA and MDS. Compared with MDS group (F), the number of fat cells (black arrow) in the AA group (C) was increased.

All MR images were sent to AW4.6 workstation (GE Healthcare). Three observers (two radiologists and a clinician) evaluated the images. Reader 1 is Z.Z., a junior radiologist with 3 years of experience in musculoskeletal MRI. Reader 2 is W.W., a senior radiologist with 22 years of experience in musculoskeletal MRI. Reader 3 is B.Y., a hematologist with no experience in musculoskeletal MRI. The image datasets of patients and controls were anonymized and presented to the observers in a random order. The gender, age, and patient information were all blinded to the readers. Each reader performed the following evaluations independently. T1‐ and T2‐weighted images were used as references for FF and R2* quantification. Regions of interest (ROIs) were manually drawn in the IDEAL‐IQ sequence FF images and R2* images of the left posterior superior iliac spine in the largest level. Red bone marrow and puncture holes were excluded (Fig. 1). The system automatically calculated the FF and R2* mean values for the drawn regions.

### Statistical Analysis

SPSS 25 software was employed for statistical analysis. Continuous variables were tested for normality and homoscedasticity. Continuous data were tested for normality with the Shapiro–Wilk test. To compare differences in FF and iron content among the three groups, the Kruskal–Wallis test was applied to non‐normally distributed data. Interobserver agreement between the three observers on parameter measurements was analyzed by calculating the interclass correlation coefficient (ICC). ICCs were interpreted as follows: 0–0.20, poor agreement; 0.21–0.40, fair agreement; 0.41–0.60, moderate agreement; 0.61–0.80, good agreement; and 0.81–1.00, excellent agreement. Area under the receiver operating characteristic (ROC) curve (AUC) analysis was performed to identify the optimal FF threshold to differentiate AA from MDS combined controls. Specificity and sensitivity were calculated according to the cut‐off value that maximized the Youden index. A *P*‐value of <0.05 indicated a statistically significant result for all tests.

## Results

A total of 118 patients were recruited, including 22 patients with MDS, 72 patients with AA, and 24 patients in the control group. Sixteen patients were excluded due to prior bone marrow transplantation, including 15 patients with AA and 1 patient with MDS. Finally, the study included 21 patients with MDS (9 males, age range: 38–77 years, median: 56 years), 57 patients with AA (29 males, age range: 20–69 years, median: 42 years), and 24 control subjects (15 males, age range: 26–68 years, median: 51 years). The age of AA patients was significantly lower than that of MDS patients and control subjects. The MDS and control groups were not significantly different in terms of age (*P* = 0.542). There were no significant differences in sex ratio (*P* = 0.250) and body mass index (BMI) (*P* = 0.776) between the AA and MDS groups.

**TABLE 1:** Comparison FF and iron content of AA, MDS, and control subjects

	AA (*N* = 57)	MDS (*N* = 21)	Control (*N* = 24)	*P*/*t* (AA‐MDS)	*P*/*t* (AA‐control)	*P*/*t* (MDS‐control)
FF (%)	79.46 ± 15.00	42.78 ± 30.09	65.50 ± 14.73	<0.001/5.258	0.001/3.733	0.439/−1.452
Age (years)	42.25 ± 14.39	58.05 ± 12.99	51.67 ± 13.24	<0.001/−4.002	0.032/−2.554	0.542/1.339
R2* (/second)	171.13 ± 100.89	145.38 ± 53.33	135.99 ± 32.41	0.553^a^		

^a^ *P*‐values for comparison between the AA, MDS, and control.

**FIGURE 2:**
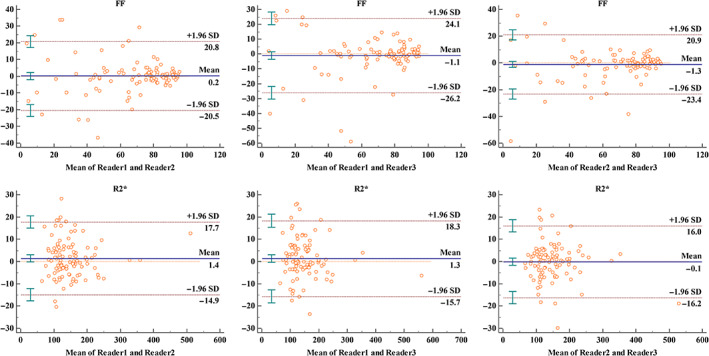
Bland–Altman analysis of three observers for FF and R2* values.

Image acquisition and data analysis were successful in all participants. The ICC of FF values were 0.978 (95% confidence interval [CI]: 0.968–0.985) between reader 1 and reader 2, 0.967 (95% CI: 0.951–0.977) between reader 1 and reader 3, and 0.977 (95% CI: 0.966–0.985) between reader 2 and reader 3, indicating near‐perfect interobserver agreement (all *P* < 0.05). The ICC of R2* values was 0.978 (95% CI: 0.968–0.985) between reader 1 and reader 2, 0.980 (95% CI: 0.970–0.986) between reader 1 and reader 3, and 0.970 (95% CI: 0.956–0.980) between reader 2 and reader 3, indicating near‐perfect interobserver agreement. Applying Bland–Altman statistics, it indicated a reliable interobserver agreement between observers. The corresponding Bland–Altman plots for three observers are shown in Fig. 2.

**FIGURE 3:**
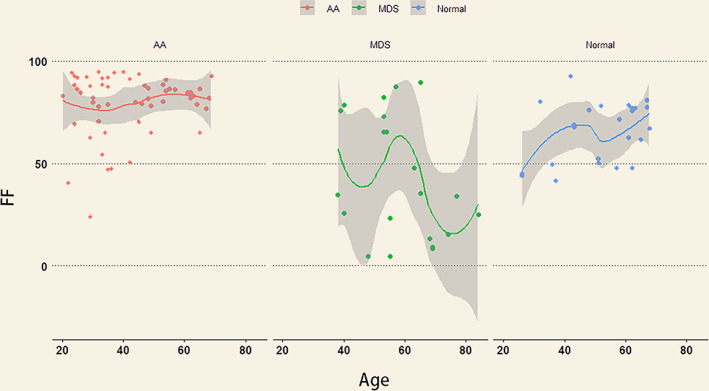
Scatterplots of FF against age for AA, MDS, and control groups. The line represents best‐fit regression trend. The shaded area is CI. The FF values in the AA group were higher than in the MDS and control groups.

The FF of patients with AA (79.46% ± 15.00%) was significantly higher than that of patients with MDS (42.78% ± 30.09%) and of control subjects (65.50% ± 14.73%). The FF in control subjects was not significantly different to that in patients with MDS (*P* = 0.439). There was no significant difference (*P* = 0.553) in the R2* values of patients with MDS (171.13 ± 100.89/second), AA (145.38 ± 53.33/second), and control groups (135.99 ± 32.41/second; table 1). Scatterplots of FF against age for AA, MDS, and control groups are shown in Fig. 3.

**FIGURE 4:**
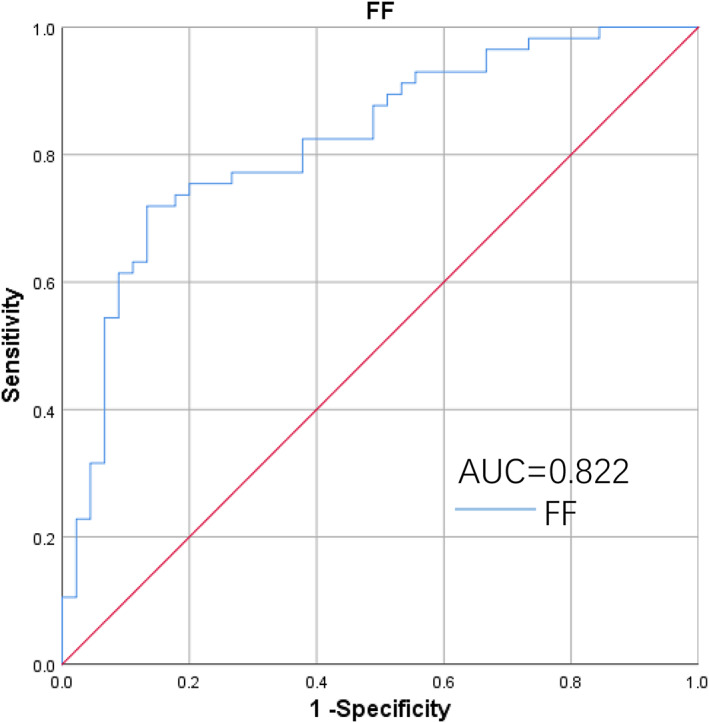
ROC curve of FF for differentiating AA with MDS combined control group.

Using ROC analysis, the area under the curve value of FF was 0.822, the sensitivity was 0.719, and the specificity was 0.867. The critical value of FF for AA was 78.67. AUC value of age was 0.266 (Fig. 4).

## Discussion

This study used IDEAL‐IQ imaging to measure FF and R2* for hemopathy. We found a significantly elevated ilium FF in AA compared to MDS and normal bone marrow. MDS showed no significant difference in FF from control subjects. This result is consistent with the current clinical diagnosis of AA, which mainly relies on bone marrow biopsy to detect the fat content. However, the IDEAL‐IQ method has the potential to assess this noninvasively. There was a significant difference between the age of patients with AA and MDS. However, through ROC curve analysis, age did not affect the diagnosis of AA and MDS. Differentiation of these two diseases also needs to be combined with blood examination. Peripheral blood smears of controls would be expected to be normal, while those of MDS patients would not. The iron content of the bone marrow, as reflected by the R2* values, is no different among AA patients, MDS patients, and control subjects. AA and MDS patients with severe hemocytopenia often need blood transfusion, and multiple blood transfusions lead to increased iron deposition in various tissues.^26^ Blood transfusion can cause iron deposition in organs, but not in the bone marrow. IDEAL‐IQ imaging can also quantify iron deposition in other tissues^21^, ^22^ and has promise in monitoring the occurrence of adverse reactions.

In our study, the ICCs of our independent readers indicated near‐perfect interobserver agreement, regardless of years' experience or medical specialty (radiologist/clinician). These results suggest that the determination of FF and R2* appears to be reader‐independent and that it does not require extensive training.

T1‐ and T2‐weighted image could not quantify FF. MR spectroscopy assessed fat content is impractical in some clinical settings due to long scan time, small imaging range, and a substantial amount of postprocessing.^14^ Positron emission tomography/computed tomography (PET/CT) examination has shown low bone metabolism in AA patients, which could be used for differential diagnosis from atypical MDS.^27^ However, PET/CT is expensive and requires injections of radioactive agents. Quantitative analysis of bone marrow fat (BMF) content through IDEAL‐IQ imaging allows for the detection of abnormal changes in the bone marrow. In combination with the clinical manifestations and relevant laboratory tests, it has the potential to improve the diagnostic accuracy of AA and MDS. A correct diagnosis based on noninvasive examination may avoid the need for invasive procedures.

In addition to monitoring AA, IDEAL‐IQ imaging may also be useful for monitoring disease progression of MDS and acute myelocytic leukemia, which typically is induced by genetic mutations leading to cloned hematopoiesis.^28^, ^29^, ^30^ Other predisposing factors for MDS and AA include apoptosis induced by DNA damage from cytotoxic chemotherapy, radiotherapy, chemical or physical drugs, and clinical drugs (chloramphenicol, nonsteroidal anti‐inflammatory drugs, antiepileptic drugs, and antithyroid drugs).^31^ IDEAL‐IQ may have potential for monitoring BMF change during and after treatment to prevent the occurrence of AA or MDS.

Bone marrow perfusion is correlated to BMI in adults. The intramedullary blood flow and the exchanges between bone marrow and bone vessels increase with BMI.^32^ In our study, there was no statistically significant difference in BMI between the three groups in this study. The effect of BMI on measuring bone marrow FF was excluded. The technique using standard T1‐weighted images was less sensitive to variations in fat concentration in the bone marrow.^33^ However, IDEAL‐IQ can sensitively detect variations in fat concentration in the bone marrow. The IDEAL‐IQ technique has a shorter scan time and can provide much greater coverage. The IDEAL method may therefore be a better choice than MR spectroscopy and the standard T1‐weighted method when assessing BMF for clinical and research purposes.^33^, ^34^ More recent work has revealed that BMF plays an important role in many diseases. BMF accumulation is thought to be correlated with endocrine and metabolic diseases, such as obesity, osteoporosis, aging, and type 1 diabetes.^35^, ^36^ It has also been associated with bone metastases in cancer.^37^, ^38^, ^39^, ^40^ In addition, bone marrow is closely related with hematopoietic function.^41^ The decrease of hematopoietic activity in bone marrow with age may be related to the accumulation of BMF.^41^, ^42^ IDEAL‐IQ imaging can be used for quantitative measurement of BMF, and may be of value for the assessment and diagnosis of diseases, such as iron deposition in liver after blood transfusion, sacroiliitis, and diabetes.^15^, ^16^, ^17^, ^18^ Although BMF content can be accurately measured, more studies are needed with comparisons to pathological results to demonstrate increased applications of IDEAL‐IQ.

### Limitations

This study was performed at a single center on a small number of subjects. Studies including larger patient cohorts in multicentric trials will be necessary to further demonstrate the robustness of IDEAL‐IQ results. The bone marrow can also be affected by various factors and individual differences. This leads to the discrete curve of FF in patients with MDS in the experiment. In addition, to keep ROIs consistent with the bone marrow biopsy, only one level of the left posterior superior ilium was selected in this experiment, not the whole pelvis.

## Conclusion

IDEAL‐IQ technology can efficiently quantify the content of fat and iron in bone tissue. The values of FF determined by IDEAL‐IQ were able to differentiate between AA and MDS without the need for an invasive procedure.

## Conflict of Interest

The authors have no conflict of interest to declare.
